# Cognitive functioning in patients with neuro-PASC: the role of fatigue, mood, and hospitalization status

**DOI:** 10.3389/fneur.2024.1401796

**Published:** 2024-06-27

**Authors:** Joshua Cahan, John-Christopher A. Finley, Erica Cotton, Zachary S. Orban, Millenia Jimenez, Sandra Weintraub, Tali Sorets, Igor J. Koralnik

**Affiliations:** ^1^Northwestern Medicine, Davee Department of Neurology, Chicago, IL, United States; ^2^Feinberg School of Medicine, Northwestern University, Chicago, IL, United States; ^3^Mesulam Center for Cognitive Neurology and Alzheimer’s Disease, Chicago, IL, United States; ^4^Northwestern Medicine, Department of Psychiatry and Behavioral Sciences, Chicago, IL, United States

**Keywords:** Long COVID, COVID-19, post-acute sequelae of SARS-CoV-2 infection (PASC), cognitive, hospitalization, depression, anxiety, fatigue

## Abstract

This study sought to characterize cognitive functioning in patients with neurological post-acute sequelae of SARS-CoV-2 infection (Neuro-PASC) and investigate the association of subjective and objective functioning along with other relevant factors with prior hospitalization for COVID-19. Participants were 106 adult outpatients with Neuro-PASC referred for abbreviated neuropsychological assessment after scoring worse than one standard deviation below the mean on cognitive screening. Of these patients, 23 had been hospitalized and 83 had not been hospitalized for COVID-19. Subjective cognitive impairment was evaluated with the self-report cognition subscale from the Patient-Reported Outcome Measurement Information System. Objective cognitive performance was assessed using a composite score derived from multiple standardized cognitive measures. Other relevant factors, including fatigue and depression/mood symptoms, were assessed via the Patient-Reported Outcome Measurement Information System. Subjective cognitive impairment measures exceeded the minimal difficulties noted on objective tests and were associated with depression/mood symptoms as well as fatigue. However, fatigue independently explained the most variance (17.51%) in patients’ subjective cognitive ratings. When adjusting for fatigue and time since onset of COVID-19 symptoms, neither objective nor subjective impairment were associated with prior hospitalization for COVID-19. Findings suggest that abbreviated neuropsychological assessment may not reveal objective difficulties beyond initial cognitive screening in patients with Neuro-PASC. However, subjective cognitive concerns may persist irrespective of hospitalization status, and are likely influenced by fatigue and depression/mood symptoms. The impact of concomitant management of fatigue and mood in patients with Neuro-PASC who report cognitive concerns deserve further study.

## Introduction

Post-acute sequelae of SARS-CoV-2 infection (PASC), also known as “Long COVID,” is a common condition affecting millions of people in the United States. An ongoing Household Pulse Survey by the National Center for Health Statistics estimates that 17.8% of all adults in the United States have had PASC (1). The persistent symptoms of PASC involve multiple organ systems cared for by many medical specialties (2). The neurological manifestations of PASC (referred to as “Neuro-PASC”) are particularly concerning as they may involve cognitive symptoms that affect quality of life and the ability to work (3–6). Further understanding the factors that influence persistent cognitive symptoms after COVID-19 can inform risk assessment and treatment for Neuro-PASC. Several pathogenic factors have been proposed in the literature, including chronic inflammatory responses, ongoing neurovascular dysfunction, autonomic dysregulation, metabolic disturbances, impaired neurotransmission, and concomitant organ system involvement (7, 8). It is unlikely, however, that any single pathogenic factor fully explains the persistent cognitive symptoms observed in individuals with Neuro-PASC. The confluence of these pathogenic factors along with critical illness-related factors (e.g., delirium, mechanical ventilation) may confer the greatest risk of persistent cognitive dysfunction (9, 10). Given the complexity of these interrelated factors and lack of diagnostic markers and robust neuropathological data to confirm their mechanistic role, researchers have begun investigating whether surrogate markers of acute COVID-19 symptom severity are associated with persistent cognitive sequelae (11). Specifically, research has used hospitalization status as a proxy for acute COVID-19 symptom severity (11). Most extant literature has found that patients who are hospitalized for COVID-19 have a higher propensity to develop persistent cognitive symptoms (9, 12–16). Indeed, this association suggests acute COVID-19 symptom severity is an important factor for the persistent cognitive symptoms in patients with Neuro-PASC. However, there are gaps in the literature that would be helpful to expand upon to further understand the relationship between hospitalization status and persistent cognitive symptoms in patients with Neuro-PASC.

First, cognitive symptoms associated with Neuro-PASC are often described with the transdiagnostic term “brain fog” (15–22). Although this descriptor captures a wide range of symptoms, it is typically indicative of deficits in attention, working memory, processing speed, and problem-solving, collectively referred to as “frontal network dysfunction” (23–25). Frontal network functions—predominately those associated with processing speed, attention, working memory, and set shifting—have been reported to be marginally impaired after hospitalization due to COVID-19 (13, 26). Other studies, including one involving >80,000 participants (15), have reported that in addition to these cognitive difficulties, memory encoding is worse in post-hospitalization patients compared to those who have not been hospitalized. It is important to note that some cognitive symptoms may change over time following hospitalization (3). For example, prior research has found that language difficulties diminish more quickly than attention difficulties post-hospitalization (24). Thus, the duration of time between COVID-19 infection and cognitive assessment should be considered when investigating the relationship between hospitalization status and cognitive functioning, which has been overlooked in some studies [for review, see (3)].

Although “brain fog” and “frontal network dysfunction” are widely referenced in the literature, they are largely based on studies using brief screening tools, such as the Montreal Cognitive Assessment or Mini Mental Status Examination (3, 27). These screeners may not adequately capture the cognitive deficits associated with Neuro-PASC and hospitalization status (28). The few extant studies assessing multiple other cognitive domains report mixed findings (13, 29), suggesting the severity of dysfunction varies according to the type of cognitive abilities being assessed. Furthermore, most studies assessing “brain fog” in Neuro-PASC have not focused on objective measures alongside subjective ones. To our knowledge, only one study has examined both persistent subjective and objective cognitive difficulties following COVID-19 and found no association between the two (29). Nevertheless, subjective and objective cognitive symptoms, when measured in isolation across different studies, are independently associated with hospitalization status in patients with Neuro-PASC (3, 9). Because subjective and objective measures may assess different aspects of cognitive functioning (30), using them interchangeably could yield variable findings.

Second, among the limited studies assessing cognition post-hospitalization, even fewer have considered additional risk factors that may affect the relationship between cognition and hospitalization status. Fatigue and depression/mood symptoms are among the most commonly identified risk factors for Neuro-PASC (31), and are associated with cognitive dysfunction (32). These factors may also influence the association between cognitive functioning and hospitalization status (29, 33). In fact, some research suggests that subjective cognitive symptoms are more closely associated with fatigue, pain, and mood issues than are objective symptoms following COVID-19 (33). Because these factors are modifiable, it would be helpful to determine if they influence the association between hospitalization status and both subjective and objective cognitive functioning.

Third, existing studies have investigated hospitalization status and cognitive functioning in patients evaluated for various subjective cognitive and non-cognitive concerns following COVID-19. These patients are often screened for objective cognitive symptoms that warrant further assessment by specialists. Yet, no study has exclusively focused on patients who undergo additional assessment due to seeming difficulties on cognitive screening (e.g., scoring ≥1 SD below normal population average). Studying this population is particularly relevant for healthcare professionals because it focuses on patients who undergo testing that entails more than a screening measure, allowing for interrogation of impairments beyond frontal network dysfunction. The discrepancy between subjective reports and expanded objective measurement of cognition noted above further highlights the need to study this subpopulation.

With these gaps outlined, it is important to acknowledge that even though cognitive screening may not adequately assess cognitive dysfunction, recommending patients to undergo comprehensive neuropsychological assessment, which requires several hours of standardized objective testing in addition to subjective cognitive assessment, may not be feasible or necessary. For this reason, healthcare systems have begun referring patients who are flagged on cognitive screening for abbreviated neuropsychological assessments to help determine the indication for cognitive rehabilitation (34). These triaged assessments may utilize a select battery of standardized tests to further characterize patients’ cognitive difficulties beyond what is indicated on cognitive screening without requiring lengthy testing procedures. Investigating the relationship between cognitive functioning and COVID-19 hospitalization status in patients undergoing abbreviated neuropsychological assessments would help clinicians understand not only the link between persistent symptoms and hospitalization, but also the utility of these assessments in further characterizing potential cognitive difficulties.

The current study sought to address these gaps by (1) further characterization of cognitive functioning and (2) examination of the relationship between cognitive functioning and hospitalization status in patients with Neuro-PASC referred for abbreviated neuropsychological assessment due to below average performance on cognitive screening. Cognitive functioning was assessed using multiple objective and subjective measures and scores were adjusted for relevant factors, including time since infection, fatigue, and co-occurring depression/mood symptoms. Hospitalization status was used as a proxy for acute COVID-19 symptom severity, as done in prior research (9, 12–16).

## Materials and methods

### Participants

A subset of 106 consecutive patients were selected from a prior study (9) investigating hospitalization status in a larger Neuro-PASC sample. Exclusion criteria for this prior study were limited to the absence of any neurological symptoms. Patients with preexisting medical or neurological conditions were not excluded since the study findings aimed to represent the neuropsychiatric functioning of patients who receive treatment in a neurology clinic. Of the individuals who were selected from this prior study, 23 had been hospitalized and 83 had not been hospitalized for COVID-19. Patients were included in the current study if they had (1) scored ≥1 SD below the mean on ≥1 selected screening measures (i.e., Pattern Comparison Processing Speed, Flanker Inhibitory Control and Attention, Dimensional Change Card Sort, and List Sorting Working Memory Test) from the National Institute of Health (NIH) Toolbox General Cognition battery (v2.1; 35); (2) symptoms consistent with COVID-19 as per Infectious Diseases Society of America guidelines; (3) confirmed SARS-CoV-2 infection via positive reverse transcription polymerase chain reaction or rapid antigen test from a nasopharyngeal swab, and/or positive SARS-CoV-2 antibody test conducted prior to COVID-19 vaccination; (4) ≥1 neurological symptoms persisting for ≥12 weeks since COVID-19 symptom onset; and (5) complete data.

### Procedures

Patients underwent an abbreviated neuropsychological assessment involving record review, clinical interview, and administration of a fixed neurocognitive test battery at a Midwestern academic medical center between 2020 and 2022. Patients were referred for this assessment if they were seen in a neurology COVID-19 clinic at the same medical center and scored ≥1 SD below the mean on any NIH Toolbox cognitive screener, which was completed on average 6 months following their COVID-19 symptom onset. The majority of assessments were conducted remotely versus in person by a board-certified behavioral neurologist (JC) or clinical neuropsychologist (EC). The prior study utilizing data from some of these patients found no differences in NIH Toolbox cognitive test scores between those who were evaluated remotely versus in person. Data were collected from all aspects of the assessment procedures, including the neurocognitive testing, interview, and record review. This study received prior approval by Northwestern University institutional review board for research as part of a larger study investigating the neurological correlates of COVID-19 (STU00212583).

### Measures

#### Subjective cognitive impairment

Subjective cognitive impairment was measured via the computerized adaptive test (CAT) version of the Patient Reported Outcome Measurement Information System (PROMIS) Cognitive Function scale (2.0) (36). The CAT version of this scale automatically chooses from a bank of 32 items depending on the participant’s responses. Each question is self-rated using a five-point Likert scale to assess perceived difficulties within the past week. Total PROMIS ratings are expressed as T-scores (ranging from 10 to 90 with a mean of 50 and standard deviation of 10), which are referenced against a normative sample in the United States. Lower *T*-scores indicated greater perceived impairment.

#### Objective cognitive performance

Objective cognitive performance was measured via a standardized composite of scores from seven performance measures from our fixed battery. The battery and normative data for the measures were based on the phone-based Uniform Data Set v3.0 from the National Alzheimer’s Coordinating Center (37). This included the Montreal Cognitive Assessment (assessing global cognition), Craft Story Recall (assessing immediate and delayed recall of verbal information), Verbal Fluency Test (assessing semantic and lexical fluency), and Oral Trail Making Test Part B (assessing complex attention). Participants were also administered the Boston Naming Test-15 Item (assessing confrontation naming); but we did not include these scores in our composite score because no norms exist for this test. Instead, we list the Boston Naming Test-15 scores in Table 1 for descriptive purposes. For the other measures, raw scores were transformed into *z*-scores adjusted for age, sex, and education according to the Uniform Data Set norms. Lower *z*-scores indicated worse performance. To remain statistically powered, we averaged the (non-weighted) *z*-scores to produce one index of objective performance.

**Table 1:** Sample demographics and clinical characteristics.

	Post-hospitalization group (*n* = 23)	Non-hospitalized group (*n* = 83)	Effect sizes (Cramér’s *V*/Cohen’s *d*)
Age	*M* = 55.26 (*SD* = 12.77)	*M* = 45.30 (*SD* = 12.75)	0.78^**^
Sex: Female	11 (48%)	63 (76%)	0.25^**^
Racial identity			
White	16 (70%)	65 (78%)	0.06
Black	3 (13%)	8 (10%)	0.01
Asian	1 (0%)	1 (1%)	0.00
Other	3 (13%)	9 (11%)	0.00
Years of education	*M* = 15.65 (*SD* = 2.01)	*M* = 16.08 (*SD* = 2.43)	0.19
Intubated during Hospitalization	5 (22%)	–	–
Subjective cognitive impairment (*T*-scores)	*M* = 33.57 (*SD* = 6.89)	*M* = 32.76 (*SD* = 6.16)	0.13
Objective cognitive performance (*Z*-scores)	*M* = −0.66 (*SD* = 0.87)	*M* = −0.76 (*SD* = 0.75)	0.14
MoCA total score (*Z*-scores)	*M* = −0.57 (*SD* = 1.19)	*M* = −0.83 (*SD* = 0.88)	0.26
Lexical fluency (*Z*-scores)	*M* = −1.02 (*SD* = 0.84)	*M* = −1.02 (*SD* = 0.98)	0.00
Semantic fluency (*Z*-scores)	*M* = −0.41 (*SD* = 0.97)	*M* = −0.70 (*SD* = 0.91)	0.31
Immediate memory (*Z*-Scores)	*M* = −0.71 (*SD* = 1.19)	*M* = −1.01 (*SD* = 1.03)	0.28
Delayed memory (*Z*-Scores)	*M* = −0.81 (*SD* = 1.24)	*M* = −1.26 (*SD* = 1.09)	0.41
Oral trail making test part B (*Z*-scores)	*M* = −0.84 (*SD* = 2.22)	*M* = −0.12 (*SD* = 1.95)	0.36
Boston naming test 15-item (Raw scores)	*M* = 13.65/15 (*SD* = 1.53)	*M* = 14.06/15 (*SD* = 1.57)	0.41
Internalizing Psychopathology (*T*-scores)	*M* = 59.75 (*SD* = 5.84)	*M* = 60.72 (*SD* = 6.49)	0.11
Fatigue (*T*-scores)	*M* = 65.65 (*SD* = 9.97)	*M* = 66.22 (*SD* = 8.28)	0.30
Time since COVID-19 Infection (Days)	*M* = 355.39 (*SD* = 190.95)	*M* = 379.96 (*SD* = 224.46)	0.16

*N* = 106; M, Mean; SD, Standard deviation; MoCA, Montreal Cognitive Assessment.

^*^p < 0.05; ^**^p < 0.01.

#### Mood, fatigue, and time since infection

Self-reported fatigue was assessed via the CAT version of the PROMIS Fatigue scale (1.0). The CAT version of this scale chooses from a bank of 95 items and uses a five-point Likert scale to assess symptoms within the past week. Scores were expressed as *T*-scores, ranging from 10 to 90 (36). Self-reported depression/mood symptoms were assessed via the CAT version of the PROMIS Anxiety and Depression scales (1.0), which chooses from a bank of 29 items for Anxiety and 28 items for Depression using the same Likert scale and *T*-score ranges as the other PROMIS scales described above, assessing symptoms within the past week (36). For this study, scores were expressed as the average of the *T*-scores from these scales. Higher T-scores for fatigue and depression/mood symptoms ratings indicated greater symptom severity. Time since infection was the number of days between COVID-19 symptom onset and neuropsychological assessment.

### Statistical analysis

Assumptions were met and *post-hoc* power analyses indicated findings had ≥80% observed power. Differences in demographics and characteristics were compared between post-hospitalized and non-hospitalized patients using independent samples *t*-tests and chi-square tests, as appropriate. To investigate group differences in subjective and objective cognitive functioning, we first conducted multiple independent samples *t*-tests. To gain a clearer understanding of the breakdown in objective cognitive performance, we conducted independent *t*-tests for each cognitive test. However, the objective composite score was used instead of each test in the subsequent analyses. We then ran separate linear regression analyses to determine whether fatigue, mood, and time since infection were associated with cognitive functioning (as measured via the composite score) and hospitalization status. If these variables were significantly associated with cognition and hospitalization status, they were used as covariates in a one-way analysis of covariances. The one-way analysis of covariances assessed for group differences in cognitive functioning, while also controlling for the effects of any relevant factors. Anonymized data may be shared upon reasonable request.

## Results

As shown in Table 1, there were no significant differences (*p* > 0.05) in clinical characteristics and demographics between post-hospitalization and non-hospitalized patients, except for gender and age. Although both groups comprised patients in mid-adulthood, post-hospitalization patients were, on average, ~10 years older with a trend for fewer females. Patients overall endorsed significantly more fatigue and depression/mood symptoms than the PROMIS normative sample, but no significant group differences were found. Neuropsychological assessments were conducted on average 12.81 months post-COVID-19 symptom onset and the duration did not significantly differ between groups.

**Figure 1:**
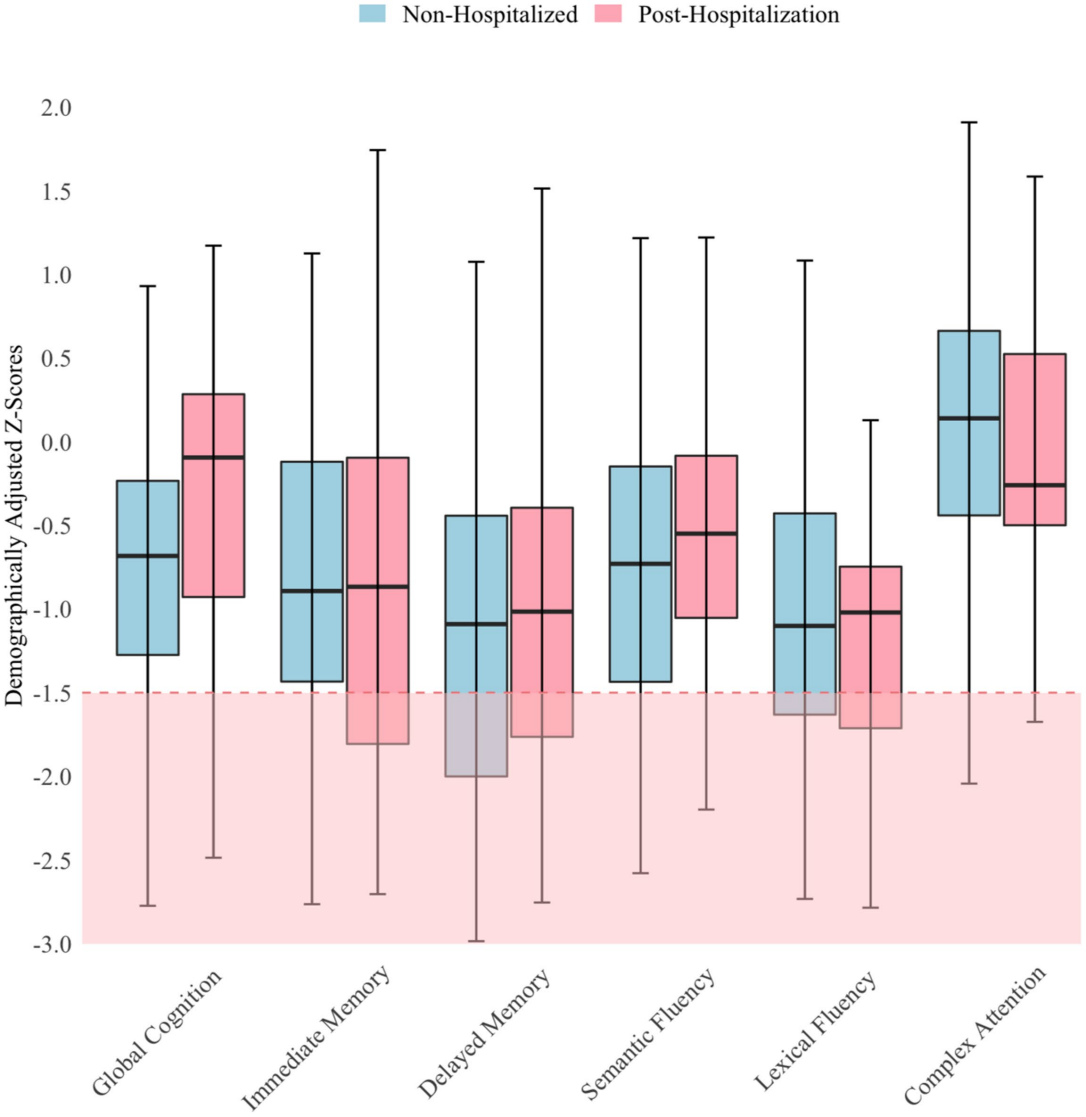
Objective cognitive performance by hospitalization status *N* = 106; Horizontal lines within the box plots represent median scores and error bars represent 95% confidence intervals.

Regression analyses indicated that neither mood, fatigue, nor time since infection were significantly associated (*p* > 0.05) with objective cognitive performance or hospitalization status. Thus, independent *t*-tests were used to compare cognitive performance between hospitalization groups, and the findings were nonsignificant. Both groups performed about one SD below the normative mean (mean *z*-score = −0.74; *SD* = 0.77) on cognitive testing. Analyses relating to objective performance were based on the composite score; but for descriptive purposes, a more nuanced illustration of patients’ performance is provided in Figure 1. As shown in Figure 1, performance did not vary much across measures, with few scores <1.5 SD below the mean. The red shaded area in Figure 1 indicates scores lower than −1.5 SD, which is considered below expectation for patients. Performance was most reduced on measures of delayed memory in the non-hospitalized group, whereas performance was most reduced on a measure of lexical fluency in the post-hospitalization group. Both groups performed best on the Oral Trail Making Test Part B, a measure of executive attention.

**Figure 2:**
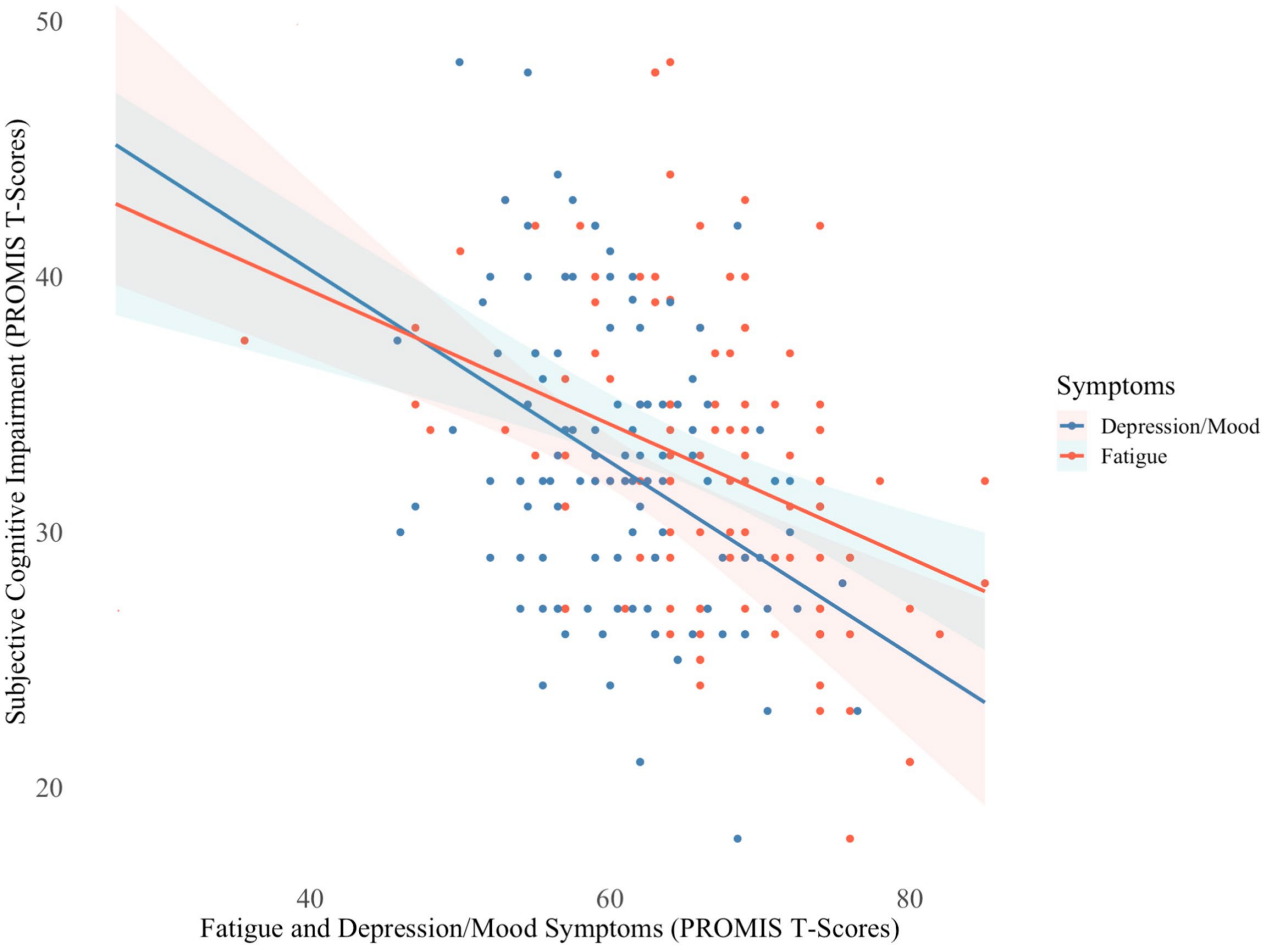
Fatigue and depression/mood symptoms in relation to subjective cognitive impairment *N* = 106; PROMIS, Patient Reported Outcome Measurement Information System. Lower T-scores indicate greater subjective cognitive impairment and higher T-scores indicate greater severity of fatigue and depression/mood symptoms.

Similarly, independent *t*-tests revealed no significant differences in subjective cognitive impairment ratings between hospitalization groups. Regression analyses indicated that greater fatigue and depression/mood symptoms were associated with greater subjective cognitive impairment (*F*[3,104] = 15.89, *p* < 0.001; *R^2^* = 34.13%), but fatigue independently explained the majority of variance (Δ*R^2^* = 17.51%, *p* < 0.001) in subjective ratings (Figure 2). To maintain parsimonious modeling, fatigue was the only covariate included in the follow-up analysis. When controlling for fatigue, however, subjective cognitive impairment still did not significantly differ between groups. Subjective cognitive impairment ratings were close to two SDs below the normative mean, implying they had significantly greater perceived cognitive difficulties than neurotypical controls.

## Discussion

This study investigated the relationship between hospitalization status and cognitive functioning in a selected group of patients with Neuro-PASC. We sought to expand upon prior research by (1) exclusively examining patients referred for abbreviated neuropsychological assessment after scoring below expectation on cognitive screening, (2) further characterizing the type and extent of cognitive dysfunction by evaluating subjective and objective cognitive functioning, and (3) considering other risk factors associated with cognitive functioning and hospitalization status.

Findings indicated that hospitalization status did not predict subjective or objective cognitive functioning in this referred patient group. These findings are not entirely surprising since the extent of variability in cognitive dysfunction is attenuated when investigating a more cognitively homogenous group. This study coupled with prior research (9), suggests the presence of cognitive difficulties may be associated with hospitalization status, but not necessarily the severity or type of difficulties. Little variability in cognitive performance was found across and within groups. Although we selected a Neuro-PASC sample enriched for potential cognitive difficulties, our assessment did not reveal more deficits than were detected on the initial cognitive screening with the NIH toolbox tests. Most patients performed within the low average range on our test battery. A few patients had below-average scores, and much fewer had exceptionally low scores (38). Although the relative difficulties on memory and lexical fluency measures and below-expectation performance on the screener may suggest some frontal networks and limbic networks dysfunction (which has also been found in prior research) (39–42), the overall scores from cognitive testing were too limited in variability and degree of impairment to pinpoint specific neural network dysfunction. Nevertheless, most patients endorsed a high degree of cognitive difficulties on self-report questionnaires. The aggregated effect size of cognitive performance (*z*-score of −0.74) in our sample indicating low average-to-average performance is consistent with prior research (13). It was somewhat unexpected that participants performed largely within normal limits on the Oral Trail Making Test Part B, given that this measure is thought to assess abilities involving frontal network functions, including executive attention and set shifting. Furthermore, research has demonstrated that patients with PASC perform poorly on the Written Trail Making Test Part B (19, 40, 43). However, the Written and Oral Trail Making Test Part B have been found to index slightly different cognitive constructs and are not considered fully convergent measures (44).

Beyond elucidating the relationship between persistent cognitive symptoms and COVID-19 hospitalization status, these specific findings carry potential implications for the referral of patients with cognitive difficulties identified through screening measures. That is, they may indicate whether such patients should be referred for comprehensive or abbreviated neuropsychological assessments, or whether no additional testing is indicated. These implications may be particularly useful for clinics using a triaged system to characterize persistent cognitive symptoms in patients with Neuro-PASC. However, additional studies administering other types of cognitive tests, especially those assessing different aspects of executive functioning, are needed to determine these important referral decisions.

The current study findings also highlight the importance of addressing fatigue and depression/mood symptoms in Neuro-PASC patients with cognitive concerns. Mood and fatigue are potentially modifiable and may contribute to perceived cognitive difficulties. Consistent with prior PASC research (13), most of our sample reported elevated levels of depression/mood symptoms and fatigue. When compared to the broader Neuro-PASC population (9), our cohort reported comparable levels of fatigue, but increased depression/mood symptoms in the post-hospitalization group. Depression/mood symptoms and fatigue are thought to have cognitive mediating effects after COVID-19 (3). Mood disturbances are frequently observed as a consequence, contributing factor, or mitigating element in various neuro-medical conditions. In those with Neuro-PASC, new onset mood disturbances may be indicative of limbic and frontal network dysfunction (39–42). Although the current study was not designed to elucidate the mechanisms underlying the association between depression/mood symptoms, fatigue, and subjective cognitive impairment, prior research has identified several putative mechanisms (3). These mechanisms include viral persistence in the nervous system, neuroinflammation that compromises blood–brain barrier integrity, cerebral microvascular injury, autoimmunity, and mitochondrial dysfunction (3). The complex and potentially overlapping nature of neural networks involved in mood, fatigue, and subjective cognition may render them susceptible to this wide range of pathogenic factors and insults (39, 40, 42). However, mood and fatigue symptoms may also be premorbid, due to psychosocial factors unrelated to COVID-19, or health-related stress from non-neurological PASC symptoms. However, our findings suggest that the relationship between mood, fatigue, and cognition depends on whether cognition is measured subjectively or objectively. It should be noted that because our sample was clinically referred and thus enriched for mood dysfunction, there was more homogeneity across hospitalization groups than observed in prior studies (that found differences in cognition), which may have further attenuated the differences in cognition between groups.

Self-report measures indicating more difficulty than is observed on objective cognitive testing is not unique to Neuro-PASC. This discrepancy has been attributed to depression/mood and somatic symptoms involving fatigue and pain in mixed clinical populations (30). Others have proposed this discrepancy exists because of the limited ability to detect subtle, yet meaningful changes in cognitive functioning with standardized tests (45). Addressing cognitive concerns is important regardless of objective performance as they may interfere with quality of life and influence patients to seek additional treatment (46).

The current study findings should be interpreted with the understanding that our small post-hospitalization subsample evaluated within a single academic medical center limits generalizability. Although our findings revealed an association between subjective cognitive impairment, depression/mood symptoms, and fatigue, we cannot determine whether such associations are causal. Further prospective research designs are needed to elucidate potentially causal relationships. A related limitation was the imbalance in the number of participants between groups, which should be addressed in future research by including larger and more balanced groups. Another limitation was using a single score to index subjective and objective cognitive difficulties. This approach may have convoluted the association between hospitalization status and specific types of cognitive symptoms (e.g., working memory vs. delayed memory). However, it seems unlikely that specific types of deficits on objective cognitive testing were driving the overall relationship, as Figure 1 does not indicate that one cognitive domain was particularly impaired. We were unable, however, to discern which types of symptoms were most impaired within the single measure used to index subjective cognitive impairment. Another potential limitation was that we did not conduct formal validity testing to help establish the validity of patients’ test scores; but it is unlikely any patients were exaggerating performance on cognitive testing given that no one performed in the exceptionally low range on any tests, and no one failed the empirical verbal fluency embedded validity indicator (47). Using multiple embedded validity indicators may be most useful to include in these types of abbreviated assessments since they can adequately detect invalid performance without adding time or costs (48, 49). The final limitation is that hospitalization status is an imperfect proxy for acute COVID-19 symptom severity. It is possible that some non-hospitalized patients may have experienced severe symptoms considering the availability of hospital beds varied across hospitals during the pandemic. However, we do not think that this is very likely since our hospital network was never overwhelmed.

As new SARS-CoV-2 variants emerge, COVID-19 continues to occur despite vaccination and boosters. In this setting, Neuro-PASC will likely remain a debilitating illness affecting people’s quality of life and ability to work (1). Thus, it is important to further characterize and identify factors that influence the persistent cognitive symptoms after COVID-19. This study further investigated these cognitive symptoms and potential contributory factors in patients clinically referred for an abbreviated neuropsychological assessment. Findings suggest that abbreviated neuropsychological assessment may not reveal objective difficulties beyond initial cognitive screening. However, cognitive concerns may persist irrespective of hospitalization status, and are likely influenced by fatigue and depression/mood symptoms. Treating providers should therefore be attuned to the association between cognition, fatigue, and depression/mood symptoms. Studies focusing on combined management of those Neuro-PASC manifestations are warranted to maximize treatment outcomes.

## Data availability statement

The raw data supporting the conclusions of this article will be made available by the authors, without undue reservation.

## Ethics statement

The studies involving humans were approved by Northwestern University Feinberg School of Medicine. The studies were conducted in accordance with the local legislation and institutional requirements. The participants provided their written informed consent to participate in this study.

## Author contributions

JC: Conceptualization, Methodology, Writing – original draft, Writing – review & editing. J-CF: Conceptualization, Methodology, Writing – original draft, Writing – review & editing, Formal analysis. EC: Conceptualization, Writing – review & editing. ZO: Data curation, Writing – review & editing. MJ: Data curation, Writing – review & editing. SW: Conceptualization, Writing – review & editing. TS: Writing – review & editing. IK: Conceptualization, Investigation, Methodology, Project administration, Writing – review & editing.
